# O037. Should aircrafts never land? Headache attributed to aeroplane travel: a new series of 140 patients

**DOI:** 10.1186/1129-2377-16-S1-A166

**Published:** 2015-09-28

**Authors:** Federico Mainardi, Ferdinando Maggioni, Carlo Lisotto, Giorgio Zanchin

**Affiliations:** Headache Centre, Neurological Division, SS Giovanni e Paolo Hospital, Venice, Itay; Headache Centre, Department of Neurosciences, Padua University, Padua, Italy; Headache Centre, S. Vito al Tagliamento Hospital, S. Vito al Tagliamento, Italy

## Background

Following our previously published paper on 75 cases [1], a new form of headache, Headache attributed to aeroplane travel (AH), has been recently codified in the ICHD-3beta classification [2].

## Materials and methods

Since our publication, we continued to receive worldwide filled-in questionnaires.

## Results

Up to now, 140 cases (males: 59%) were studied. A strictly unilateral side was reported in 85% of patients; side-shift in different attacks was observed in 21%. The pain site was mainly frontal-orbital (n=110) or frontal-parietal (n=9). The mean age at onset was 35.9 years (range 7-63). AH attacks occurred during landing (in nine patients also during take-off), lasted less than 30 minutes and remitted spontaneously. Its intensity was very severe or severe. Only in 16 cases the first attack occurred during the first flight. The attacks presented in more than 50% of flights in 38 patients; 24 reported its occurrence during every flight. AH negatively affected the propensity to air travel in more than 75% of the sufferers. Prophylactic use of NSAIDs prevented or effectively relieved the attacks in more than 50% of cases.

## Conclusions

Considering the impact of AH, passengers should be appropriately informed about the existence of this severe headache, on its benign nature and its potential prevention. These new data confirm the stereotyped features of this specific headache, in keeping with the ICHD criteria.

Written informed consent to publish was obtained from the patient(s).
